# Prevalence of *Salmonella* in juvenile dogs affected with parvoviral enteritis

**DOI:** 10.4102/jsava.v89i0.1731

**Published:** 2018-12-05

**Authors:** Willem J. Botha, Johan P. Schoeman, Stanley L. Marks, Zandri Whitehead, Cornelius H. Annandale

**Affiliations:** 1 Department of Companion Animal Clinical Studies, University of Pretoria, South Africa; 2 Department of Medicine and Epidemiology, University of California, United States; 3 Department of Production Animal Studies, University of Pretoria, South Africa

## Abstract

Salmonellosis is a disease of major zoonotic importance and canine parvovirus is a potentially fatal cause of canine enteritis with a world-wide distribution. Persistent isolation of Salmonella during routine environmental sampling surveys of a hospital ward, reserved for the treatment of dogs with canine parvovirus infection, prompted investigation into a possible source. We hypothesised that dogs affected by canine parvovirus would have a higher prevalence of faecal salmonellae compared to an apparently healthy cohort. Seventy-four client-owned dogs naturally infected with canine parvovirus and 42 apparently healthy client-owned dogs were included in the study. This prospective, longitudinal, observational study was conducted over an 18-month period. Fresh faecal samples were collected from dogs aged 6 weeks to 9 months diagnosed with canine parvovirus infection and admitted for treatment, and from apparently healthy dogs presented for vaccination or routine hospital procedures. Faeces were submitted for the isolation, antimicrobial susceptibility testing and serotyping of salmonellae. The prevalence of faecal Salmonella shedding was 22% and 31% for the affected and apparently healthy dogs, respectively, which was not statistically different. No significant associations between Salmonella status and possible risk factors or continuous variables such as age, body weight and duration of hospitalisation were identified. All the Salmonella isolates (*n* = 32) were resistant to penicillin G, lincomycin and tylosin. Salmonellae from nine different serotypes were identified. The prevalence of Salmonella shedding in both groups was higher than that commonly reported, yet similar to those in previous reports on young dogs, shelter dogs or dogs fed a raw meat diet.

## Introduction

Canine parvovirus (CPV) is a universally prevalent and potentially fatal cause of canine enteritis, which is often exacerbated by concurrent enteropathogen infections (Prittie 2004). Salmonellosis is a well-established major zoonotic disease, which is commonly acquired as a foodborne disease in humans mostly through faecally contaminated food (Behravesh et al. 2010; Imanishi et al. 2014). Zoonotic transmission of *Salmonella* within a veterinary practice and outbreaks of salmonellosis in both large and small animal facilities have also been reported (Cherry et al. 2004; Hartmann et al. 1996; Ketaren et al. 1981). In addition, *Salmonella enterica* subsp. *enterica* is also a well-recognised nosocomial pathogen in large-animal veterinary hospitals (Timoney, Neibert & Scott 1978). Animals have been infected with *S. enterica* via oral exposure under experimental conditions, but the circumstances predisposing to and promoting transmission under natural conditions remain unclear. As a consequence, it is important to evaluate the risk factors that increase the likelihood of infection (Grassl & Finlay 2008). Following an outbreak of salmonellosis in the large-animal section of the Onderstepoort Veterinary Academic Hospital, infection control measures were initiated, and in-hospital environmental niches of *Salmonella* were identified and controlled. However, the persistent isolation of *Salmonella* during follow-up of microbiological surveys targeting the isolation ward, dedicated to the treatment of dogs infected with CPV, prompted further investigation into this cohort of patients being a possible source of contamination.

The prevalence of salmonellae amongst diarrhoeic dogs in South Africa is unknown. This study was designed to determine the comparative prevalence of *Salmonella* shedding in juvenile dogs infected with CPV and a cohort of apparently healthy age-matched control dogs.

## Materials and methods

### Study overview

This was a prospective, longitudinal, observational study performed on client-owned dogs. Juvenile dogs, diagnosed with CPV infection and admitted to the Onderstepoort Veterinary Academic Hospital for treatment, were sampled as an affected cohort. Clinically healthy dogs within a matching age-range, presented for routine hospital visits, were sampled as an apparently healthy cohort.

The study was conducted over the course of 18 months from October 2015 to March 2017. Dogs were only entered into the study with informed consent from their owners.

### Animal information

Sample size calculations performed at an approximate prevalence of *Salmonella* shedding in diarrhoeic dogs of 5% (Marks et al. 2011) with a precision level of 5% and 95% confidence interval suggested that a minimum number of 73 dogs with CPV infection should be collected. As a lower prevalence was expected in apparently healthy dogs, it was aimed to collect approximately one healthy dog for every two CPV-affected dogs.

Dogs were included in the affected cohort if they were (1) aged between 6 weeks and 9 months, (2) diagnosed with CPV infection using a commercial quick enzyme-linked immunosorbent assay (IDEXX CPV SNAP, IDEXX Laboratories, Westbrook, ME, United States [US]) which was confirmed by electron microscopic identification of the virus in faeces, with supporting clinical signs such as inappetence, vomiting and diarrhoea, and (3) were admitted for treatment to the isolation ward.

Dogs were included in the apparently healthy cohort when they presented for (1) vaccinations, blood donor screening, ovariohysterectomy and orchiectomy, (2) were aged between 6 weeks and 9 months of age and (3) were deemed clinically healthy based on anamnesis and full clinical examination.

Dogs were excluded from the study, in either cohort, if they had received antibiotic therapy at any stage throughout their lives prior to presentation. In addition, dogs from the apparently healthy cohort were excluded from the study if they had any history suggestive of previous or current illness, or if CPV was detected on electron microscopy of faeces.

### Sampling

On presentation, data were collected regarding several historical and clinical variables, that is, the dietary history of the dog, current or recent (< 1 month) antibiotic use by any humans or animals within the household shared by the dog and previous visits of the animal to either a veterinary or human hospital. The diet was recorded as being home-cooked, commercial (store-bought), premium (veterinary retail only) or mixed. The remainder of the questions was only answered as ‘yes’ or ‘no’.

Fresh faecal specimens from both cohorts were collected on admission by using either a sterile 1 mL syringe or a gloved finger inserted into the rectum. The faecal specimens were submitted to the Bacteriology Laboratory, Department of Veterinary Tropical Diseases and Electron Microscopy Unit, Department of Anatomy and Physiology, Faculty of Veterinary Science, University of Pretoria, in capped 1 millilitre (mL) syringes (diarrhoeic specimens) or Eppendorf tubes (formed stool). Canine parvovirus shedding from the infected cohort (CPV ELISA SNAP positive dogs) was confirmed via negative-staining transmission electron microscopy (Philips CM 10 transmission electron microscope, Philips Electron Optical Division, Eindhoven, The Netherlands). Faecal specimens from the apparently healthy cohort were submitted for negative-staining transmission electron microscopy to exclude faecal shedding of CPV. Canine parvovirus ELISA SNAP tests were not performed on this cohort.

### Culture of Salmonella enterica subsp. enterica

Faeces were submitted for the selective isolation of *Salmonella* using a previously reported technique (Lyle et al. 2015). The recovered isolates were stored at -70 °C in brain–heart infusion broth, pending serotyping using typing antisera at a reference laboratory (General Bacteriology Laboratory, Agricultural Research Council, Onderstepoort Veterinary Institute, Onderstepoort, South Africa).

The submitted faecal specimens were incubated in an enrichment broth of buffered peptone water at 37 °C for 24 hours. The specimens were then vortexed, and 1 mL incubated in a selective tetrathionate broth with brilliant green (TBG) for a further 24 h at 43 °C. An aliquot (0.1 mL) of vortexed TBG was then transferred to 10 mL of Rappaport-Vassiliadis broth (RV) and incubated for 24 h at 43 °C after which a vortexed sample was plated onto xylose-lysine-tergitol (XLT4) agar. After overnight incubation at 43 °C, suspect colonies (pink colonies with or without black centres) were plated onto Columbia blood agar plates and incubated for 24 h at 37 °C. *Salmonella enterica* isolation was confirmed by biochemical testing using a commercial kit (API10S, BioMirieux, Marcy I’Etoile, Rhône-Alpes, France).

Antimicrobial susceptibility testing was performed using the Kirby-Bauer disc diffusion method (Bauer et al. 1966). Antimicrobial agents used in susceptibility testing included a standardised panel of amikacin, amoxicillin/ampicillin, doxycycline/oxytetracycline, enrofloxacin, gentamicin, penicillin G, trimethoprim/sulphamethoxazole, chloramphenicol, cephalexin/cephalothin, kanamycin, lincomycin, lincospectin, orbifloxacin, amoxicillin clavulanic acid, tylosin and polymyxin B.

Dogs from the infected cohort were treated according to a standard protocol, with adjustments as dictated by the clinical condition. In addition, data regarding the length of hospitalisation and outcome were collected and recorded. Where possible, fresh rectal faecal specimens were collected again at discharge from affected dogs. These faecal samples were also submitted for *Salmonella* isolation.

### Statistical analysis

Results were entered into an Excel (Microsoft Excel, Microsoft Corporation, Redmond, WA, US) spreadsheet. Continuous data were assessed for normality using Shapiro-Wilk testing and descriptive statistics was calculated using a statistical software package (IBM SPSS Statistics Version 24, Chicago, IL, US). The Chi-Square or Fisher’s exact tests were performed to test for significance between proportions as required by the specific data sets. A 5% level of significance was considered statistically significant for all comparisons.

### Ethical considerations

The study was approved by the animal ethics committee of the University of Pretoria (V091-15).

## Results

The study comprised 74 CPV-infected dogs and 42 apparently healthy dogs. The prevalence of *Salmonella* shedding was 22% (16/74) and 31% (13/42) in infected and apparently healthy dogs, respectively, and the difference was not significant (*P* = 0.26).

The infected cohort comprised 45% (33/74) female and 55% (41/74) male dogs and the apparently healthy cohort comprised 62% (26/42) female and 38% (16/42) male dogs. There was no significant difference in sex ratio between the groups (*P* = 0.07), nor was there any association between sex and the isolation of *S. enterica* (*P* = 0.623). The median age of both the infected and apparently healthy cohorts was 3 months (range = 6 weeks to 8 months). The median body weight for the infected and apparently healthy cohorts was 5.9 kg (range = 0.8 kg – 30.8 kg) and 6.3 kg (range = 1.9 kg – 22.0 kg), respectively. Neither age (*P* = 0.241) nor body weight (*P* = 0.223) were significantly associated with the isolation of *S. enterica*. Both cohorts comprised various breeds with the most common being mixed breed (19%, 14/74), American Pitbull terriers (15%, 11/74) and Boerboels (14%, 10/74). The rest of the breeds included six Jack Russell terriers, five Dachshunds, four Belgian Malinois Shepherds, four Staffordshire Bullterriers, three Rottweilers, three Yorkshire terriers, two Labrador Retrievers, and one each of the following breeds: Maltese terrier, Miniature Pinschers, Border Collie, German Shepherd, Golden Retriever, Pekingese, Pomeranian, Pug, Rhodesian Ridgeback, Scottish terrier and Siberian Husky.

Of the CPV-infected cohort, 3% (2/74) of dogs were fed home-cooked diets, 68% (50/74) commercial diets, 1% (1/74) premium diets and 28% (21/74) mixed diets. None of the dogs in the apparently healthy cohort were fed a home-cooked diet, and 45% (19/42) were fed commercial, store-bought diets, 33% (14/42) premium, veterinary-specific diets and 22% (9/42) mixed diets. Eleven per cent (8/74) of owners in the CPV-infected cohort indicated that antibiotics were being used at home at the time of presentation with none reporting antibiotic use in the apparently healthy cohort. Fifty-nine per cent (44/74) of the CPV-infected cohort and 22% of the apparently healthy cohort reported prior visits to a veterinary practice. The nature of these visits was not recorded for every individual. Of the 74 CPV-infected dogs, 3% (2/74) had three vaccinations, 16% (12/74) had two vaccinations, 35% (26/74) had a single vaccination and 46% (34/74) had never been vaccinated. There was no significant association between the type of diet fed (*P* = 0.335), antibiotic use in the home environment (*P* = 0.483), previous hospital visits (*P* = 0.678) or previous vaccinations (*P* = 0.177) and the isolation of *S. enterica*.

Thirty-eight per cent (28/74) of the CPV-infected cohort had a follow-up faecal specimen collected at discharge and *S. enterica* was isolated from 7% of dogs (2/28). One dog was positive for the isolation of *S. enterica* on both the admission and discharge specimen, and the second dog was positive on the discharge specimen only. In addition, three dogs that were positive for *S. enterica* at admission were negative at discharge.

The mortality rate (18%; 13/74) in the CPV-infected cohort was similar to that previously reported in the literature in general and from the same institution, in particular (Goddard et al. 2008; Schoeman, Goddard & Leisewitz 2013; Schoeman & Herrtage 2008). Five dogs were euthanised owing to poor prognosis and eight dogs died naturally. The median hospitalisation duration was 5 days (range = 2–11). No significant association between isolation of *S. enterica* and length of hospitalisation (*P* = 0.72) or survival (*P* = 0.328) was identified in the CPV-infected cohort.

All *S. enterica* subsp. *enterica* isolates (*n* = 32) were resistant to penicillin G, lincomycin and tylosin. Nine of the isolates were resistant to lincospectin and 21 showed intermediate (*n* = 20) or complete resistance (*n* = 1) to doxycycline/oxytetracycline. All the isolates were sensitive to amikacin, amoxicillin/ampicillin, enrofloxacin, gentamicin, trimethoprim sulphamethoxazole, chloramphenicol, cephalexin/cephalothin, orbifloxacin, amoxicillin clavulanic acid and polymixin B.

Four serotypes were identified amongst the 32 isolates of *S. enterica*. The serotype of 16 isolates could not be determined by the reference laboratory and seven could only be partially serotyped. The serotyping results are listed in Table 1.

**TABLE 1:** Serotypes of *Salmonella* spp. recovered from 32 faecal isolates of juvenile dogs co-infected with canine parvovirus and an apparently healthy cohort of age-matched controls.

Serotype	Number of isolates
S. Heidelberg 4,5:r:1,2	4
Salm II 18:z10:z6	4
S. Chile 6,7:z:1,5	2
S. Cotia 18:-:1,6	2
S. Braenderup 6,7,14:e,h:e,n,z1	1
Salm II 4,5:z:1,5	1
Salm II 16:z:e,n,x	1
Salm II 30:b:z6	1
Salm Poly OMD	16

Note: *Salmonella* spp. serotypes as identified by a reference laboratory using commercial antisera. Salm Poly OMD isolates could not be completely serotyped and Salm II isolates were only partially identified.

## Discussion

This study is the first to report the prevalence of *Salmonella* shedding in a cohort of CPV-infected dogs, and it identified a prevalence of *S. enterica* of 22% in CPV-infected dogs and 31% in apparently healthy dogs. The comparative prevalence of *S. enterica* shedding was not statistically different between the dogs with parvoviral enteritis and an age-matched clinically healthy cohort.

The reported prevalence of *S. enterica* in diarrhoeic dogs is extremely variable, ranging between 0% and 76% (Khan 1970; Seepersadsingh, Adesiyun & Seebaransingh 2004; Stone et al. 1993). However, most non-diarrhoeic dogs ingesting processed commercial diets have a prevalence of *S. enterica* below 4.4% (Adesiyun, Campbell & Kaminjolo 1997; Seepersadsingh et al. 2004; Shimi, Keyhani & Bolurchi 1976; Timbs et al. 1975). Higher prevalences have been reported in dogs housed in a shelter, stray populations, working dogs used at an abattoir or on farms, hunting dogs and dogs ingesting raw meat diets (Frost et al. 1969; Khan 1970; Shimi et al. 1976; Stucker et al. 1952). One study reported a prevalence of 25% in dogs younger than 6 months of age compared to a prevalence of 5.2% in older dogs (Förster, Holland & Tesfamariam 1974). The prevalence of *S. enterica* shedding identified in this study supports the notion that juvenile dogs have a higher prevalence of *S. enterica* but failed to identify or sanction any of the previously reported risk factors. Murine studies have shown a 100 000-fold decrease in the 50% implantation dose for *Salmonella* colonisation following the disruption of the intestinal microbiota by streptomycin treatment (Que & Hentges 1985). This would suggest that all juvenile animals may have a greater susceptibility to *Salmonella* colonisation associated with the lack of a well-established intestinal microbiota (Carter & Quin 2000). Nonetheless, CPV-infected dogs are likely to suffer from a greater degree of dysbiosis compared to healthy individuals, arguing against dysbiosis as a major reason for the higher prevalence of faecal *Salmonella* in juvenile animals, when compared to adult animals.

The reported risk factors for the isolation of *Salmonella* include contact with livestock, multiple-dog households, administration of antibiotics, hospitalisation and the feeding of raw diets or treats including raw meat and eggs (Leonard et al. 2011; Reimscheussel et al. 2017; Uhaa et al. 1988). The transmission of *Salmonella* is thought to occur most frequently via the faecal-oral route (Tanaka, Katsube & Imaizumi 1976). Interestingly, in our study, most dogs were fed solely commercial or premium pelleted diets and only 28% and 22% of dogs in the CPV-infected and apparently healthy cohorts, respectively, were fed chicken, pet mince or table scraps in addition to their staple diet. One dog was fed a diet of raw meat only and *Salmonella* was not isolated from this dog. Natural treats and chews have been implicated as a possible source of exposure to both pets and owners (Finley et al. 2006). Unfortunately, the use of these products in our population was not assessed but may serve as an additional source owing to their frequency of use in puppies. Juvenile dogs may further have increased exposure via coprophagia, contact with wildlife species and ingestion of carrion, considering their inquisitive nature.

The persistent isolation of *Salmonella* during targeted environmental surveillance of the isolation ward suggested that the population of dogs housed in this environment may be a persistent source of environmental contamination. Contamination of this area was thought to act as a nidus of infection and consequent spread to other parts of the hospital. Moreover, salmonellosis is considered an important nosocomial disease in large-animal veterinary hospitals (Lyle et al. 2015). In this study, *S.* Heidelberg was the only serotype recovered from the environment in the large-animal section within the same facility (Lyle et al. 2015). This finding may suggest that there was no significant cross contamination between the two sections of the hospital. However, the relatively high prevalence of *Salmonella* shedding in juvenile dogs may raise concern for possible contamination by this population of patients within the small animal hospital. Further studies are needed to determine the significance of this notion, especially considering that targeted environmental surveillance for *Salmonella* may not be as stringent as that in large-animal hospitals.

All *S. enterica* isolates in this study were resistant to at least three antibiotics and the prevalence of resistance amongst these isolates was higher than that previously reported for isolates from dogs (Seepersadsingh et al. 2004). All the isolates were resistant to tylosin, lincospectin and penicillin G. Resistance to tylosin is unsurprising considering their limited efficacy against gram-negative bacteria (Kim et al. 2014). Lincospectin is not commonly used in small animal practice; hence, resistance to these antibiotics is of little clinical significance. Despite the fact that all isolates were resistant to penicillin G, no resistance was reported to other beta-lactam antibiotics commonly used in practice. In conclusion, none of these antibiotics are routinely used in the empirical treatment of suspected salmonellosis and therefore these resistance patterns are unlikely to have therapeutic implications. In contrast, a few isolates did show intermediate resistance to doxycycline, which may need to be closely monitored in the future.

*Salmonella enterica* was identified from two dogs at discharge. One of these two dogs had *S. enterica* isolated at admission and discharge, whereas the second dog had *S. enterica* isolated at discharge only. Possible explanations for the negative isolation of *S. enterica* at admission and positive isolation at discharge include sampling or isolation error or colonisation during the hospitalisation. The expected relatively low sensitivity of single sample versus multiple sample culture for the detection of *Salmonella* shedding may also contribute to this finding. The use of antimicrobials in the treatment of CPV may aid in the colonisation of *S. enterica* by transiently disrupting the normal microbiota and weakening the colonisation resistance offered by these microbes. However, in both cases, with positive *S. enterica* isolated at discharge, antibiotic therapy was in effect unsuccessful in preventing colonisation or clearing the dog from *S. enterica*, despite sensitive susceptibility patterns being reported to the antibiotics routinely used in the treatment of CPV cases. However, this phenomenon was negated by the three dogs that were positive for isolation of *S. enterica* at admission and negative for isolation of *S. enterica* at discharge. Asymptomatic dogs have been reported to intermittently shed salmonellae for up to 6 weeks or longer post-exposure, or up to 2 weeks following ingestion of a single contaminated meal, which suggests that longitudinal studies following juvenile dogs over several weeks with multiple cultures may be needed to truly elucidate the epidemiology of salmonellae in this population (Finley et al. 2007).

There were several limitations to this study. Firstly, dogs diagnosed with mild CPV infection and treated on an outpatient basis were not included in this study. Therefore, the prevalence of *Salmonella* shedding identified in this study potentially only reflects the more severely affected dogs and not the whole population of juvenile dogs infected with CPV. In addition, the study of CPV-infected dogs that were hospitalised and treated introduced a potential population bias, as only dogs that were owned by people who were able to afford the costs of hospitalisation and treatment were included in the study. Secondly, the use of a single sample for the isolation of *Salmonella* on admission may have caused some positive animals to remain undetected, as sensitivity of *Salmonella* culture is poor and typically 3–5 negative cultures are required to confirm lack of shedding in clinical settings (Marks et al. 2011). Thirdly, PCR or ELISA may have been a better modality than electron microscopy to exclude CPV shedding in the apparently healthy cohort owing to a higher sensitivity of the former tests (Schmitz et al. 2009). However, lack of availability and relatively higher expense precluded its use in this study. Lastly, the relatively small number of dogs in which *S. enterica* was isolated might have led to a type 2 error, thereby retaining a false null hypothesis, when assessing the risk factors for the shedding of this bacterial enteropathogen. In addition, even though a logistic regression model of statistical analysis may have been more appropriate for the assessment of possible risk factors for the identification of *Salmonella*, this was not performed owing to the relatively few *Salmonella*-positive animals and also it was not the primary aim of this study.

## Conclusion

The prevalence of *Salmonella* shedding in dogs with CPV infection was not statistically different from that in a healthy cohort. However, the prevalence in both groups was considerably higher than that previously reported (0.0% – 3.6%) for non-diarrhoeic dogs, yet similar to that reported for young dogs, shelter dogs or dogs fed a raw diet (25% – 69%). To the authors’ knowledge, this is the first report of the prevalence of *Salmonella* shedding in dogs diagnosed with canine parvoviral enteritis.
